# Supplementary material for: Human listeners’ perception of behavioural context and core affect dimensions in chimpanzee vocalizations
Source: Proc Biol Sci. 2020 Jun 17;287(1929):20201148. doi: 10.1098/rspb.2020.1148 (PMC7329049; doi:10.1098/rspb.2020.1148)
Supplement: Supplementary Materials [file rspb20201148supp1.docx]

**Supplementary Materials**

Human Listeners’ Perception of Behavioural Context and Core Affect Dimensions in Chimpanzee Vocalisations

**Text S1: Recording of chimpanzee vocalisations**

The behavioural contexts as well as classifications based on arousal level (high, medium, low) and valence (positive, negative) were determined by author K.E.S., who is an expert on chimpanzee vocal communication. K.E.S. recorded the stimuli from individuals in the Sonso community of wild chimpanzees, Budongo Forest, Uganda, and captive chimpanzees housed at Edinburgh Zoo and the Wolfgang Kohler Primate Research Centre, Leipzig, Germany. Audio recordings were made with a Sennheiser K6/ME67 directional microphone using either (i) a Sony TCD-D8 portable digital audiotape (DAT) recorder (Budongo/Edinburgh), and the recordings were then digitized at a sampling rate of 44.1 kHz, 16 bits accuracy with Cool Edit Pro LE (1999), or (ii) a MARANTZ PMD660 solid state recorder (sampling rate of 44.1 kHz, 16 bits accuracy) (Leipzig). The behaviour of the caller, the response of individuals in the group or party, and the general behavioural context were noted for each vocalisation. Only vocalisations from single individuals whose identities were known used in this study.

**
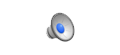
Audio 1S: Examples of chimpanzee vocalisations produced in 10 behavioural contexts**


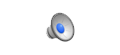
Rough grunts while eating high value food:


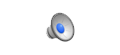
Rough grunts while eating low value food:


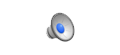
Copulation calls while having sex:


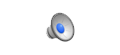
Whimpers by juveniles when separated from mother:


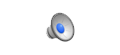
Pant hoots when discovering a large food source:


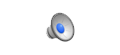
Tantrum screams when refused access to food:


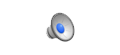
Laughter while being tickled:


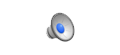
Victim screams when attacked by another chimpanzee:


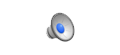
Waa barks while threatening an aggressive chimp or predator:

Alarm calls when discovering something scary:

Examples of chimpanzee vocalisations produced in 10 behavioural contexts can also be listened from <https://emotionwaves.github.io/chimp/>

**Table 1S**

Table 1S. *Comparison of accuracy in early and late trials in Experiment 1*

|  | **Percentage accuracy (Mean and SD)** | | **Pairwise comparison (first 30 trials vs last 30 trials)** | |
| --- | --- | --- | --- | --- |
|  | **First 30 trials** | **Last 30 trials** | ***Z*** | ***p-value*** |
| **Behavioural Context** | 10.30 (0.06) | 10.22 (0.07) | 0.560 | 0.576 |
| **Arousal level** | 46.74 (0.10) | 44.92 (0.11) | 2.552 | 0.011 |
| **Valence** | 51.84 (0.12) | 53.50 (0.14) | -1.898* | 0.059 |

*Note.* * t value. Wilcoxon signed-rank test is used for pairwise comparisons of accuracy in context categorisation and arousal judgements since data were not normally distributed while pair sampled t-test is used for comparisons of valence judgements since data was normally distributed.

**Table 2S**

Table 2S. ﻿*Acoustic analysis of vocalisations of each behavioural context, arousal level and valence, as Means per stimulus category (separate rows), with Standard Deviations (separated by comma)*

|  | *SCoG* | *No. Call* | *Dur.* | *f_0_ min* | *f_0_ max* | *f_0_ mean* | *f_0_ Sd* | *Time Max* | *Peak Pos.* | *%Voi* | *Jitter* | *Shimmer* | *HNR min* | *HNR max* | *HNR mean* | *HNR Sd* |
| --- | --- | --- | --- | --- | --- | --- | --- | --- | --- | --- | --- | --- | --- | --- | --- | --- |
| Eating high value food | 1161.640, 355.521 | 2.737, 0.452 | 0.142, 0.050 | 633.064, 169.215 | 800.896, 197.138 | 734.491, 186.646 | 56.475, 40.446 | 0.155, 0.054 | 114.753, 30.428 | 97.668, 5.599 | 0.000, 0.000 | 1.442, 0.179 | -225.78, 0.524 | 28.907, 6.067 | 11.499, 3.149 | 5.579, 0.895 |
| Eating low value food | 482.756, 400.873 | 3.545, 1.011 | 0.94, 0.030 | 462.106, 153.080 | 551.556, 192.803 | 501.649, 164.476 | 36.217, 31.461 | 0.143, 0.029 | 171.512, 53.826 | 55.094, 27.668 | 0.002, 0.005 | 2.064, 0.428 | -224.97, 0.363 | 21.095, 5.874 | 1.462, 1.697 | 4.490, 0.908 |
| Copulating (having sex) | 951.698, 511.915 | 2.273, 0.905 | 0.261, 0.180 | 711.059, 210.167 | 806.838, 236.922 | 757.436, 217.221 | 27.300, 15.048 | 0.184, 0.165 | 73.575, 31.598 | 100.000, 0.000 | 0.000, 0.000 | 1.356, 0.241 | -226.02, 0.465 | 29.355, 5.330 | 13.803, 3.073 | 5.730, 1.062 |
| Being separated from mother | 1148.748, 740.622 | 3.700, 1.06 | 0.242, 0.087 | 583.082, 169.378 | 693.122, 256.831 | 632.062, 191.137 | 32.205, 28.531 | 0.185, 0.052 | 96.676, 42.150 | 96.928, 6.314 | 0.000, 0.000 | 1.465, 0.236 | -225.58, 0.384 | 23.626, 6.196 | 9.010, 5.097 | 4.328, 0.958 |
| Discovering a large food source | 1653.112, 617.530 | 3.833, 0.937 | 0.568, 0.237 | 282.587, 116.743 | 771.236, 157.211 | 561.161, 142.385 | 154.175, 85.360 | 0.444, 0.193 | 90.054, 17.448 | 95.582, 4.925 | 0.001, 0.001 | 1.323 0.248 | -226.068, 0.409 | 33.819, 4.702 | 12.974, 4.726 | 6.645, 1.114 |
| Being refused access to food | 3000.796, 493.814 | 2.200, 0.414 | 0.375, 0.099 | 877.821, 355.987 | 1408.327, 242.432 | 1200.024, 234.628 | 185.684, 143.729 | 0.308, 0.115 | 81.464, 16.294 | 87.578, 22.024 | 0.000, 0.000 | 1.626, 0.248 | -225.446, 0.319 | 25.390, 7.112 | 5.824, 2.806 | 3.780, 0.816 |
| Being tickled | 1437.125, 1344.207 | 4.750, 1.915 | 0.172, 0.113 | 374.973, 250.662 | 610.146 298.147 | 456.149, 242.061 | 92.842, 117.532 | 0.213, 0.096 | 136.606, 47.763 | 60.033, 33.584 | 0.002, 0.002 | 1.778, 0.150 | -225.527, 0.481 | 25.515, 5.684 | 3.882, 3.165 | 3.384, 0.895 |
| Being attacked by another chimpanzee | 2226.703, 604.872 | 1.762, 0.625 | 0.535, 0.237 | 979.130, 383.364 | 1430.974, 172.694 | 1229.112, 247.389 | 149.64, 183.116 | 0.371, 0.156 | 74.056, 20.606 | 99.350, 1.317 | 0.000, 0.001 | 1.497, 0.252 | -225.686, 0.368 | 27.364, 6.464 | 9.559, 3.670 | 4.407, 1.423 |
| Threatening an aggressive chimp or predator | 982.113, 306.565 | 1.375, 0.500 | 0.314, 0.70 | 476.619, 197.657 | 893.497, 220.929 | 716.981, 199.027 | 127.861, 68.784 | 0.237, 0.051 | 77.019, 17.915 | 84.816, 17.448 | 0.001, 0.001 | 1.795, 0.237 | -225.720, 0.468 | 29.947, 6.239 | 6.386, 2.250 | 4.754, 1.197 |
| Discovering something scary | 758.891, 146.810 | 1.385, 0.506 | 0.441, 0.197 | 429.200, 139.269 | 630.338, 158.077 | 535.946, 130.238 | 59.533 36.618 | 0.289, 0.186 | 67.681, 23.510 | 100.000, 0.000 | 0.000, 0.001 | 1.140, 0.324 | -225.681, 0.613 | 33. 100, 7.566 | 16.294, 4.198 | 6.339, 1.674 |
| High Arousal | 1486.734, 767.927 | 1.98, 1.123 | 0.465, 0.217 | 599.327, 378.920 | 996.704, 370.779 | 822.327, 357.173 | 126.006, 122.581 | 0.333, 0.167 | 76.580, 20.895 | 95.006, 10.905 | 0.001, 0.001 | 1.465, 0.349 | -225.768, 0.474 | 30.483, 6.752 | 10.813, 5.115 | 5.335, 1.652 |
| Medium Arousal | 1577.933, 1070.613 | 3.140, 1.457 | 0.231, 0.136 | 635.323, 291.384 | 875.722, 375.458 | 763.576, 329.320 | 83.904, 104.569 | 0.209, 0.113 | 103.249, 40.778 | 87.312, 24.282 | 0.001, 0.001 | 1.546, 0.252 | -225.665, 0.480 | 26.725, 6.322 | 8.590, 4.925 | 4.551, 1.320 |
| Low Arousal | 482.756, 400.873 | 3.550, 1.011 | 0.941, 0.030 | 462.106, 153.080 | 551.556, 192.803 | 501.649, 164.476 | 36.217, 31.461 | 0.143, 0.029 | 171.512, 53.826 | 55.094, 27.668 | 0.002, 0.005 | 2.064, 0.428 | -224.974, 0.363 | 21.095, 5.874 | 1.462, 1.697 | 4.490, 0.908 |
| Positive Valence | 1094.538, 862.970 | 3.650, 1.359 | 0.208, 0.204 | 458.973, 214.835 | 672.916, 238.367 | 567.173, 213.366 | 75.184, 81.349 | 0.215, 0.146 | 133.578, 50.969 | 75.004, 29.944 | 0.001, 0.003 | 1.699, 0.410 | -225.517, 0.600 | 26.484, 7.201 | 6.789, 5.784 | 4.908, 1.451 |
| Negative Valence | 1717.861, 931.710 | 1.960, .965 | 0.400, 0.189 | 703.538, 363.699 | 1074.62556, 401.490 | 914.284, 362.193 | 120.928, 130.640 | 0.291, 0.140 | 78.081, 24.670 | 93.685, 14.174 | 0.000, 0.001 | 1.520, 0.330 | -225.632, 0.435 | 28.016, 7.223 | 9.229, 5.008 | 4.680, 1.490 |

*Note.* Mean and standard deviations of the acoustic parameters. *No. Call* = number of calls in the stimulus (mean), *Dur.* = total duration of calls in whole stimulus (seconds), *f*_0_ = fundamental frequency, *Time* *Max.* = time of the maximum peak frequency within a call; *Peak Pos.* = relative position of the peak frequency in the call; *% Voi* = percentage of voiced frames in the stimulus, *HNR* = harmonics-to-noise ratio, *SCoG* = spectral centre of gravity

**Table 3S**

|  | PCA1  (% of variance = 23.51) | PCA2  (% of variance = 22.60) | PCA3  (% of variance = 16.42) |
| --- | --- | --- | --- |
| *ScoG* | -0.67 | 0.599 | 0.388 |
| *duration* | 0.262 | 0.246 | 0.790 |
| *f_0_  min* | -0.006 | 0.901 | -0.187 |
| *f_0_  max* | 0.094 | 0.870 | 0.392 |
| *f_0_ mean* | 0.078 | 0.956 | 0.152 |
| *f_0_ Sd* | 0.062 | 0.092 | 0.735 |
| *Time Max* | 0.094 | 0.087 | 0.887 |
| *Peak Pos* | -0.514 | -0.434 | -0.242 |
| *% Voi* | 0.658 | -0.255 | 0.072 |
| *Jitter* | -0.018 | -0.250 | 0.025 |
| *Shimmer* | -0.641 | 0.033 | -0.162 |
| *HNR min* | -0.780 | 0.094 | -0.031 |
| *HNR max* | 0.781 | -0.035 | 0.153 |
| *HNR mean* | 0.887 | -0.024 | 0.054 |
| *HNR Sd* | 0.640 | -0.296 | 0.068 |

Table 3S. *Rotated component-loadings of each acoustic feature for the three-dimensional principal components’ solution*

*Note.* Bold type indicates parameters with highest loadings.

**Table 4S_A**

Table 4S_A. GLMMs testing the prediction of behavioural context, arousal and valence recognition from acoustic features

| **Fixed Effect** | **Behavioural Context** | | | | **Arousal** | | | | **Valence** | | | | |
| --- | --- | --- | --- | --- | --- | --- | --- | --- | --- | --- | --- | --- | --- |
|  | **Estimate** | **SE** | **Z Value** | **Pr (>\|z\|)** | **Estimate** | **SE** | **Z Value** | **Pr (>\|z\|)** | **Estimate** | **SE** | **Z Value** | **Pr (>\|z\|)** |  |
| Intercept | 0.669 | 0.89 | 7.495 | <0.001 | -0.08 | 0.170 | -0.518 | 0.604 | 0.251 | 0.91 | 2.738 | 0.006 |  |
| *SCoG* | 0.353 | 0.54 | 6.586 | **<0.001**** | 0.104 | 0.019 | 5.325 | **<0.001** | 0.232 | 0.019 | 11.958 | **<0.001** |  |
| *duration* | 0.158 | 0.056 | 2.825 | **<0.01*** | 0.055 | 0.019 | 2.912 | **<0.05** | 0.161 | 0.019 | 8.242 | **<0.001** |  |
| *f_0_ mean* | 0.080 | 0.462 | 1.740 | 0.082 | 0.255 | 0.019 | 13.252 | **<0.001** | 0.295 | 0.019 | 15.483 | **<0.001** |  |
| *f_0_ Sd* | -0.103 | 0.38 | -2.729 | **<0.01*** | 0.277 | 0.020 | 13.902 | **<0.001** | -0.108 | 0.019 | -5.777 | **<0.001** |  |
| *HNR mean* | -0.281 | 0.047 | -6.026 | **<0.001**** | 0.014 | 0.019 | 0.745 | 0.456 | 0 | 0.019 | 0.001 | 0.999 |  |
| *HNR max* | 0.131 | 0.040 | 3.310 | **<0.001**** | 0.015 | 0.018 | 0.811 | 0.417 | 0.003 | 0.018 | 0.186 | 0.852 |  |

*Note.* **p* < 0.05; ***p* < 0.001. Recognition scores for arousal level and valence are obtained in Experiment 1 and for behavioural context in Experiment 2. All coefficients represent changes in the log-odds of recognised or not recognised responses as a function of the acoustic predictors. ﻿Bold type indicates statistically significant p values (*p* < 0.05).

**Table 4S_B**

Table 4S_B*. Random effects in prediction of behavioural context, arousal and valence recognition*

|  |  | Context | | Arousal | | Valence | |
| --- | --- | --- | --- | --- | --- | --- | --- |
|  | | Variance | Std. Dev. | Variance | Std. Dev. | Variance | Std. Dev. |
| Part ID | | 3.284 | 1.812 | 0.071 | 0.267 | 0.176 | 0.419 |
| Chimpanzee ID | | 0.272 | 0.522 | 1.879 | 1.370 | 0.462 | 0.680 |

*Note.* Context recognition is based on context-matching task (see Experiment 2), arousal level and valance recognition are based on Experiment 1.

**Table 5S**

Table 5S. *Model selection procedure based on Akaike’s Information criterion*

|  | | Behavioural Context | | | Arousal | | | | | Valence | | | |
| --- | --- | --- | --- | --- | --- | --- | --- | --- | --- | --- | --- | --- | --- |
| Model | Intercept | | AICs | ∆AICs | | Intercept | AICs | ∆AICs | Intercept | | | AICs | ∆AICs |
| Excluding *SCoG* | 0.669 | | 17932.6 | 37.7 | | -0.097 | 56155.9 | 27.7 | 0.229 | | 54299.7 | | 142.8 |
| Excluding *duration* | 0.690 | | 17899.8 | 4.9 | | -0.082 | 56136.1 | 7.9 | 0.759 | | 54224.4 | | 67.5 |
| Excluding *f_0_* *mean* | 0.665 | | 17894.9 | **0.0** | | -0.058 | 56306.1 | 177.9 | 0.288 | | 54398.5 | | 241.6 |
| Excluding *f_0_ Sd* | 0.667 | | 17899.0 | 4.1 | | -0.070 | 56324.2 | 196 | 0.241 | | 54190.5 | | 33.6 |
| Excluding *HNR mean* | 0.638 | | 17927.7 | 32.8 | | -0.086 | 56128.2 | **0.0** | 0.251 | | 54156.9 | | **0.0** |
| Excluding *HNR max* | 0.653 | | 17902.4 | 7.5 | | -0.090 | 56128.3 | **0.1** | 0.250 | | 54157.0 | | **0.1** |

*Note.* Context recognition is based on context-matching task (see Experiment 2), arousal level and valance recognition are based on Experiment 2. Bold type indicates models with the highest power to explain variation the dependent variable (i.e., correct or incorrect response0, based on lowest AICc.
